# On the Action of Hypochlorite of Lime on Sulphur, and the Employment of a Mixture of These Bodies for the Vulcanization of India-Rubber

**Published:** 1860-07

**Authors:** Garltier de Claubry


### ARTICLE XXVI.

*On the Action of Hypochlorite of Lime on Sulphur, and the Employment of a Mixture of these Bodies for the Vulcanization of India-Rubber.* By M. Garltier de Claubry. (Comptes Rendus.)

"Mr. Parkes, of Birmingham, first made known the curious fact that caoutchouc in contact with very small quantities of chloride of sulphur dissolved in any convenient menstruum, sulphide of carbon, for example, became vulcanized at the ordinary temperature. This process allows of the vulcanization of many objects on which it would be impossible to operate at a high temperature, such as very thin sheets of caoutchouc, or woolen garments, or silk fabrics dyed in colors which cannot resist a great heat. Mr. Parkes also pointed out the precautions necessary when thick pieces are operated upon ; but whatever care may be taken, it is easy to see that it is almost impossible to obtain in this way products equally vulcanized.

"Mr. Parkes has pointed out another way by which a better effect is produced, which consists in mixing with the caoutchouc paste what he calls *dry chloride of sulphur.* This name can only be applied to flowers of sulphur impregnated with chlorine, and by means of this mixture india-rubber can really be vulcanized in the cold, and the greater part of the sulphur remain in the state of simple admixture.

"The analysis of a great number of vulcanized objects having revealed the presence of chloride of calcium, it occurred to me that this salt might come from hypochlorite of lime which had been used in the caoutchouc paste to produce the chloride of sulphur necessary for vulcanization, and that it was such a mixture which Mr. Parkes used. The following facts prove beyond doubt that it may be employed for the purpose.

"If we simply shake flowers of sulphur and dry hypochlorite of lime together at the ordinary temperature, the two are scarcely in contact before a strong smell of chloride of sulphur is manifested. If the two be rubbed together in a pestle and mortar, the temperature of the mixture rises, the sulphur becomes soft, and the whole agglutinates into mass with the abundant evolution of vapor. When the sulphur is greatly in excess relatively to the hypochlorite, the mixture of the two bodies should be made without rubbing ; it may then be added to the caoutchouc paste with or without the addition of other substances, such as chalk, zinc white, etc, and the valcanization may be effected either at the ordinary temperature, or at a gentle heat. By this process it is possible to obtain india-rubber of any thickness uniformly vulcanized.

"When sulphur is mixed with a large excess of the hypochlorite of lime by merely shaking the two together, the temperature quickly rises very high and strong action takes place, therefore the mixture must never be made in a close vessel."—*Chemical News.*

